# Vaso-Occlusive Thrombotic Ischemic Colitis Presenting With Large-Volume Hematochezia in Sickle Cell Beta^+^-Thalassemia

**DOI:** 10.14309/crj.0000000000001982

**Published:** 2026-01-22

**Authors:** Mesay Asfaw, Farshad Aduli, Maryam Homayounieh, Juan Carlos Santiago Gonzalez, Babak Shokrani, Sarrah Fadul

**Affiliations:** 1 Department of Gastroenterology and Hepatology, Howard University Hospital, Washington, DC; 2 Department of Internal Medicine, Howard University Hospital, Washington, DC; 3 Department of Pathology, Howard University Hospital, Washington, DC

**Keywords:** ischemic colitis, sickle cell disease, beta-thalassemia, vaso-occlusive crisis, gastrointestinal bleeding, hematochezia

## Abstract

We present the case of a 60-year-old African American man with sickle cell beta plus thalassemia who developed large-volume hematochezia during hospitalization for a vaso-occlusive crisis. Sickle cell disease is characterized by chronic hemolysis and vaso-occlusion that can affect the gastrointestinal tract, although clinically significant colonic ischemia remains rare. The patient had a history of diverticulosis and hemorrhoids but presented with acute lower gastrointestinal bleeding after dehydration and nonsteroidal anti-inflammatory drug exposure, both of which can impair mucosal perfusion. Colonoscopy revealed discontinuous ulcerations in the rectosigmoid colon, and histopathology confirmed ischemic colitis with thrombosed submucosal vessels, consistent with vaso-occlusive microthrombosis. This case underscores the importance of early endoscopic evaluation and recognition of ischemic colitis as a potential cause of overt gastrointestinal bleeding in patients with sickle cell disease.

## INTRODUCTION

Sickle cell disease (SCD) is a hereditary hemoglobinopathy characterized by chronic hemolysis and recurrent vaso-occlusive episodes caused by intravascular sickling, endothelial injury, and microvascular obstruction.^1^ Although vaso-occlusion most often affects the musculoskeletal and pulmonary systems, gastrointestinal involvement is well recognized but frequently underdiagnosed. Among gastrointestinal manifestations, ischemic colitis is particularly rare, with only approximately a dozen cases described in the English-language literature to date.^2–7^

The pathophysiology of colonic ischemia in SCD is driven primarily by microvascular vaso-occlusion in which sickled erythrocytes adhere to the endothelium, promote inflammation, and form intraluminal thrombi that compromise perfusion.^1^ This process may be exacerbated by dehydration, infection, or medications that impair mucosal blood flow. The rectosigmoid junction is especially vulnerable because of its limited collateral circulation and its susceptibility to hypoperfusion.^8^

Nonsteroidal anti-inflammatory drugs, which are commonly used for pain management in SCD, may compound the risk of colonic ischemia by disrupting mucosal integrity, reducing prostaglandin-mediated vasodilation, and impairing local blood flow.^3^

Because ischemic colitis in SCD is rare and symptoms such as abdominal pain or hematochezia may be attributed to more common etiologies such as hemorrhoids, diverticulosis, or an uncomplicated pain crisis, recognition can be delayed.^2,9^ In this report, we describe a patient with sickle cell beta plus thalassemia who developed large-volume hematochezia and was found to have histologically confirmed vaso-occlusive ischemic colitis. This case highlights the diagnostic challenges and management considerations associated with this underrecognized complication.

## CASE REPORT

A 60-year-old African American man with sickle cell beta plus thalassemia, complicated by thrombocytopenia and avascular necrosis, presented to the referring hospital with generalized weakness, dizziness, and diffuse pain after working outdoors in the heat. SCD complications frequently worsen with dehydration due to increased blood viscosity and sickling.^1^ He reported taking 6 tablets of nonsteroidal anti-inflammatory drugs including ibuprofen and naproxen for pain before arrival. Nonsteroidal anti-inflammatory drugs (NSAIDs) are known to impair mucosal perfusion and increase susceptibility to ischemia.^3^

**Figure 1.**
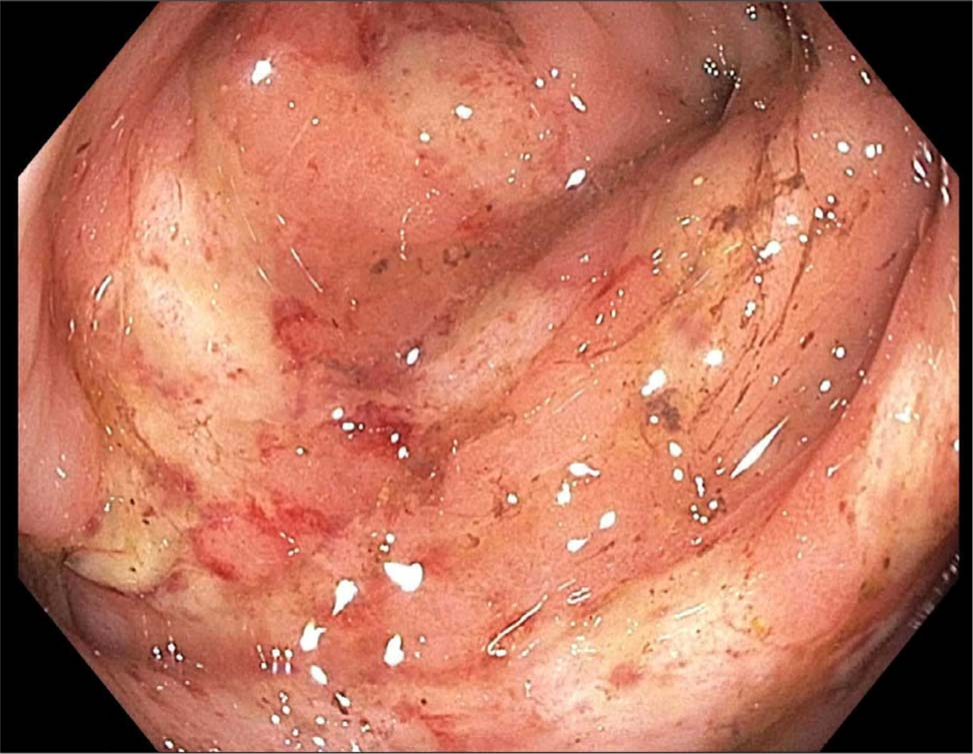
Colonoscopy, distal sigmoid colon: endoscopic image of the distal sigmoid colon showing patchy, discontinuous segments of ulcerated and erythematous mucosa without active bleeding. Biopsies were taken from these areas. Findings are consistent with ischemic type injury.

During hospitalization, he developed 2 episodes of large-volume, dark, loose bowel movements associated with urgency and lower abdominal discomfort. He also experienced acute urinary retention requiring placement of a Foley catheter (Figure 1).

Initial laboratory studies revealed hemoglobin of 6.0 g per dL, white blood cell count of 5.7 thousand per microliter, platelet count of 18,000 per microliter, lactate dehydrogenase level above 1,000 IU per L, total bilirubin 2.2 mg per dL, and elevated transaminases. After transfusion of 3 units of packed red blood cells and 1 unit of platelets, his hemoglobin rose to 8.4 g per dL and platelets to 43,000 per microliter. These values were significantly lower than Hematology clinic baseline studies 1 month earlier.

Due to ongoing concern for gastrointestinal bleeding, he was transferred to Howard University Hospital. On arrival, he was hypertensive at 162 over 128 mm Hg, tachycardic at 102 beats per minute, afebrile, and saturating well on room air. Physical examination revealed mild scleral icterus but no abdominal tenderness. Cardiopulmonary and neurologic examinations were normal.

Repeat laboratory studies demonstrated lactate dehydrogenase 1,394 IU per L, haptoglobin less than 30 mg per dL, elevated inflammatory markers, and stable renal function, consistent with ongoing hemolysis.

Cross-sectional imaging obtained before colonoscopy did not reveal evidence of mesenteric arterial or venous thrombosis, bowel obstruction, or active contrast extravasation. Absence of large-vessel occlusion supported a microvascular mechanism.^8^

**Figure 2.**
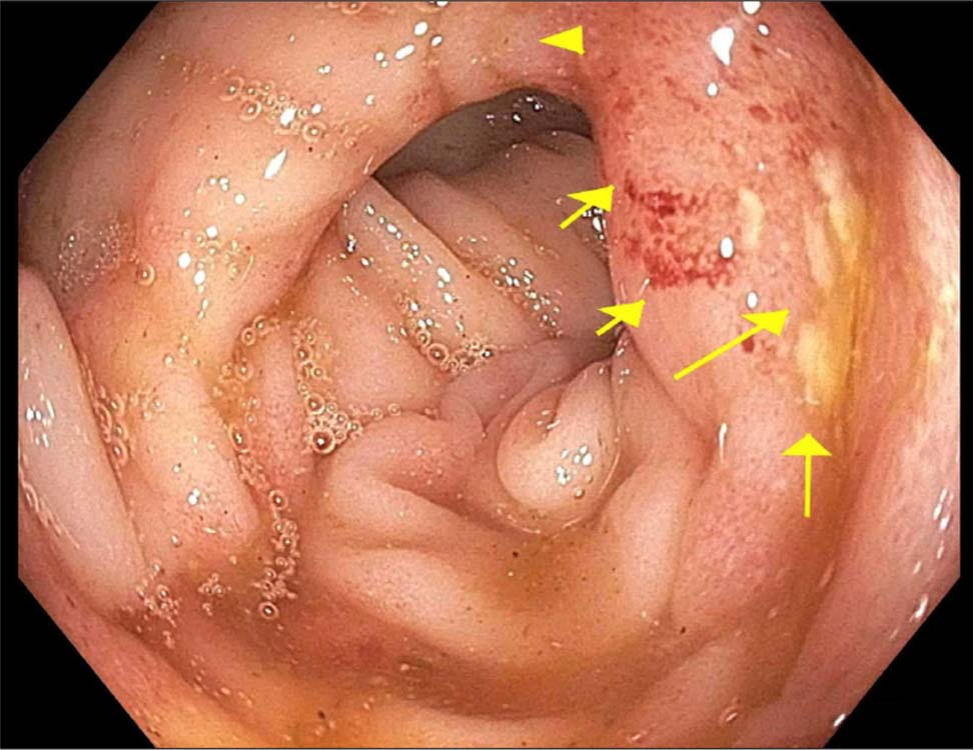
Colonoscopy, rectosigmoid region: ulcerated mucosa in the rectosigmoid region demonstrating loss of vascular pattern, edema, and shallow ulcerations. No diverticular bleeding or hemorrhoidal stigmata were present.

After stabilization with intravenous fluids, analgesia, and transfusion support, colonoscopy was performed. The examination revealed grade III internal hemorrhoids without stigmata of recent bleeding. Multiple diverticula were present in the sigmoid and descending colon but without adherent clot or evidence of active hemorrhage. The rectum and sigmoid colon contained patchy, discontinuous ulcerated mucosa without visible bleeding stigmata (Figure 2). Cold biopsies were taken from the ulcerated areas.

**Figure 3.**
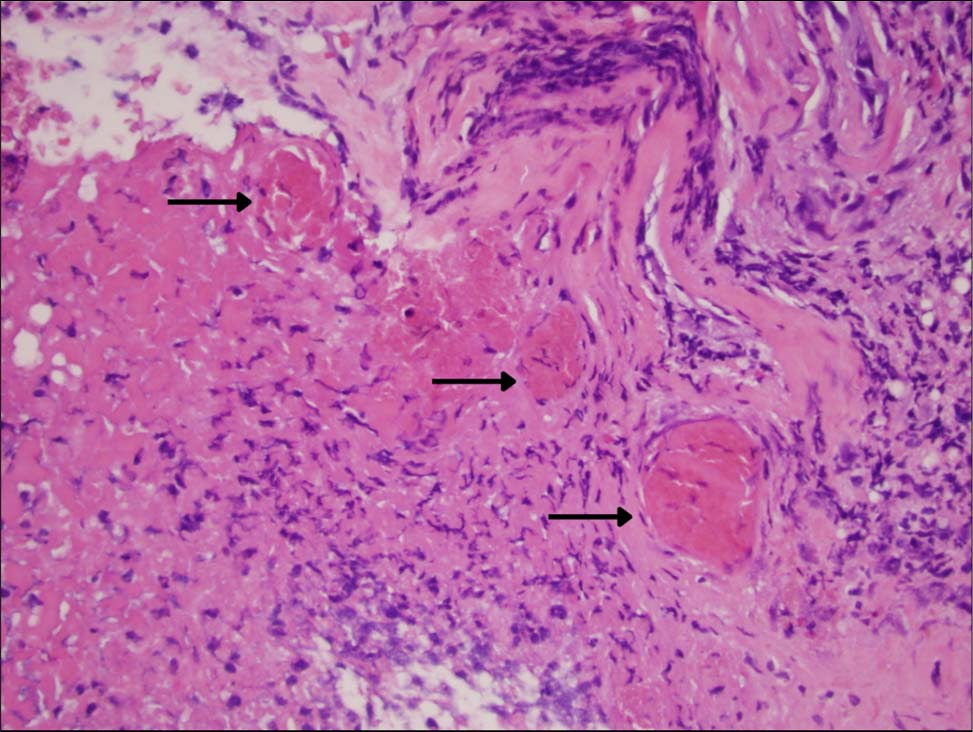
Thrombosed mucosal and submucosal vessels demonstrating superimposed acute ischemia, a characteristic histopathologic finding in patients with sickle cell disease. Hematoxylin and eosin stain, original magnification ×400.

Histopathology showed acute and chronic mucosal injury including cryptitis, crypt abscesses, mucosal ulceration, fibrin deposition, hemorrhage, pseudomembrane formation, and thrombosed submucosal vessels (Figure 3). These findings were consistent with ischemic-type colitis.^2,4–7^

## DISCUSSION

This case describes a histologically confirmed episode of ischemic colitis in a patient with sickle cell beta plus thalassemia who presented with large-volume hematochezia in the setting of a vaso-occlusive crisis, dehydration, and recent NSAID exposure. Although abdominal pain is common during vaso-occlusive episodes, clinically significant colonic ischemia is rare in SCD and may be overlooked when more common causes of lower gastrointestinal bleeding are present.^2,9^

The pathophysiology of ischemic colitis in SCD is primarily related to microvascular vaso-occlusion. Sickled erythrocytes exhibit impaired deformability and increased adhesion to the endothelium, promoting inflammation, endothelial activation, and thrombosis.^1,6^ These processes can obstruct small submucosal vessels and reduce mucosal perfusion. In our patient, symptoms occurred during a clinically recognized sickle cell crisis, laboratory studies demonstrated marked hemolysis and inflammation, and histology showed thrombosed submucosal vessels. Absence of large- or medium-vessel occlusion on imaging supported a microvascular rather than macrovascular mechanism.^8^

The differential diagnosis included diverticular bleeding and hemorrhoidal bleeding, both common causes of lower gastrointestinal hemorrhage.^9^ However, neither diverticula nor hemorrhoids demonstrated stigmata of recent bleeding on colonoscopy, and imaging showed no active extravasation. Instead, colonoscopy revealed discontinuous ulcerated mucosa typical of ischemic injury.

An additional feature of this case was the presence of both acute and chronic ischemic changes on histology. Chronic changes, including fibrosis and atrophic glands, likely represented prior subclinical episodes of ischemia, whereas acute ulceration and thrombi reflected a new injury superimposed on a chronically vulnerable mucosa. Similar layered patterns have been described in prior reports of SCD-associated ischemic colitis.^4–7^

NSAID use likely contributed to the ischemic injury by reducing prostaglandin-mediated mucosal protection and impairing local blood flow.^3^ In combination with dehydration and ongoing vaso-occlusion, NSAID exposure increased susceptibility to clinically significant colonic ischemia.

**Figure 4.**
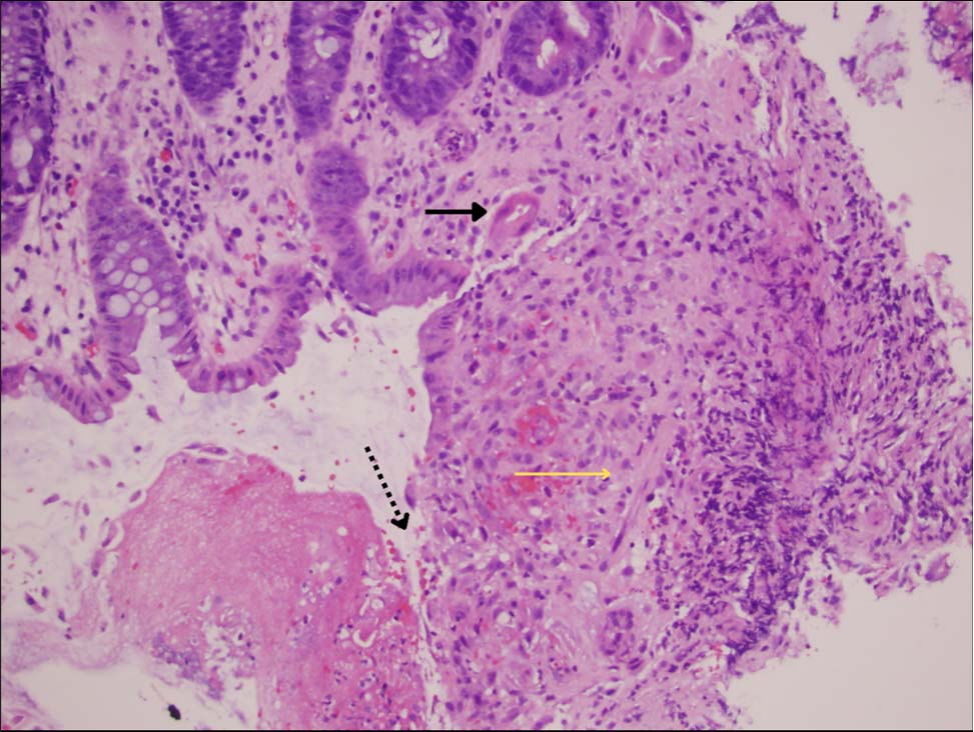
Histologic features of ischemic colitis. Solid arrow indicates atrophic, shrunken crypt glands. Dotted arrow highlights ulcerated surface epithelium consistent with acute ischemic injury. Yellow arrow denotes lamina propria fibrosis, reflecting chronic ischemia. Hematoxylin and eosin stain, original magnification ×200.

Although intestinal ischemia is well described in older adults with cardiovascular disease, it is rarely reported in patients with SCD. A review of the literature reveals approximately a dozen cases of SCD-associated ischemic colitis, spanning classic descriptions through modern case reports.^2,4–7^ These cases illustrate a range of presentations, from reversible colitis to fulminant infarction requiring surgery (Figure 4).

Early recognition is essential, as prompt fluid resuscitation, bowel rest, transfusion support, and removal of precipitating factors can prevent progression to transmural necrosis or perforation. Colonoscopy with biopsy remains the most valuable diagnostic tool when bleeding is present or when ischemia is suspected.^8^

In patients with SCD who present with overt lower gastrointestinal bleeding, ischemic colitis should be included in the differential diagnosis, particularly during vaso-occlusive crises or when additional risk factors such as dehydration or NSAID use are present.^2–7^ This case demonstrates how microvascular vaso-occlusion can lead to clinically significant colonic ischemia that may mimic more common sources of bleeding such as hemorrhoids or diverticulosis. Prompt endoscopic evaluation and histologic confirmation are essential for accurate diagnosis, and early supportive management can prevent severe complications including transmural necrosis or perforation.

## DISCLOSURES

Author contributions: M. Asfaw: Primary author, case analysis, manuscript drafting, and revision; F. Aduli and JCS Gonzalez: Case supervision and critical revision of manuscript; M. Homayounieh: Literature review and manuscript editing; B. Shokrani and S. Fadul: Histopathology interpretation, pathology figure contribution, and input on discussion section. M. Asfaw is the article guarantor.

Acknowledgments: We thank the patient and clinical staff involved in this case.

Financial disclosure: The authors declare no conflicts of interest.

Informed consent was obtained for this case report.

## ABBREVIATIONS:

dL: deciliter
GI: gastrointestinal
IU: international unit
mg: milligram
mm Hg: millimeters of mercury
NSAID: nonsteroidal anti-inflammatory drug
SCD: sickle cell disease
